# Personalized treatment based on mini patient-derived xenografts and WES/RNA sequencing in a patient with metastatic duodenal adenocarcinoma

**DOI:** 10.1186/s40880-018-0323-y

**Published:** 2018-08-23

**Authors:** Peng Zhao, Hui Chen, Danyi Wen, Shuo Mou, Feifei Zhang, Shusen Zheng

**Affiliations:** 1 Cancer Biotherapy Center, The First Affiliated Hospital of Zhejiang University School of Medicine, Hangzhou, 310003 P. R. China; 2 Department of General Surgery, Zhejiang Hospital, Hangzhou, 310013 P. R. China; 3 LIDE Biotech Co., Ltd, Shanghai, P. R. China; 4 OrigiMed Co., Ltd, Shanghai, P. R. China; 5 Division of Hepatobiliary and Pancreatic Surgery, Department of Surgery, The First Affiliated Hospital of Zhejiang University School of Medicine, Hangzhou, 310003 P. R. China; 6 Key Lab of Combined Multi-Organ Transplantation, Ministry of Public Health, Hangzhou, China

**Keywords:** Duodenal adenocarcinoma, Mini patient-derived xenograft, Whole-exome sequencing, RNA sequencing, Somatic mutation, Personalized therapy

## Abstract

**Background:**

Treatment guidelines for a variety of cancers have been increasingly used in clinical practice, and have resulted in major improvement in patient outcomes. However, recommended regimens (even first-line treatments) are clearly not ideal for every patients. In the present study, we used mini patient-derived xenograft (mini-PDX) and next-generation sequencing to develop personalized treatment in a patient with metastatic duodenal adenocarcinoma.

**Methods:**

Resected metachronous metastatic tumor tissues were implanted into SCID mice to determine the sensitivity to a variety of drug regimens. Mutation profiles were assessed using both DNA whole-exome sequencing (DNA–WES) and RNA sequencing. The results of the analyses were used to select optimal treatment for the patient with metastatic duodenal adenocarcinoma.

**Results:**

Assessment with mini-PDX models took only 7 days. The results showed high sensitivity to S-1 plus cisplatin, gemcitabine plus cisplatin and everolimus alone. The patient received gemcitabine plus cisplatin initially, but the treatment was terminated due to toxicity. The patient was then switched to treatment with S-1 alone. The overall disease-free survival was 34 months. DNA–WES and RNA sequencing identified *KRAS* mutation (A146T), *TP53* (C229Yfs*10) and *RICTOR* amplification in the metastatic duodenal adenocarcinoma. These findings provided further support to the results of the mini-PDX, and suggest mTOR inhibitors should be used if and when relapse eventually occurs in this patient.

**Conclusions:**

Mini-PDX model combined with WES/RNA sequencing can rapidly assess drug sensitivity in cancer patients and reveal key genetic alterations. Further research on this technology for personalized therapy in patients with refractory malignant tumors is warranted.

**Electronic supplementary material:**

The online version of this article (10.1186/s40880-018-0323-y) contains supplementary material, which is available to authorized users.

## Background

Duodenal adenocarcinoma is a rare tumor that accounts for less than 1% of all gastrointestinal cancers [1, 2]. Complete surgical resection is the optimal therapeutic modality for localized duodenal adenocarcinoma. Approximately 30–40% of the patients who have undergone curative resection of the primary duodenal adenocarcinoma eventually relapse [3]. In lymph node-positive patients, adjuvant chemotherapy could improve patient outcomes [4]. The addition of radiotherapy to adjuvant therapy does not confer a survival benefit in high-risk patients [5]. Due to a lack of randomized studies, optimal adjuvant chemotherapy remains a matter of debate.

In recent years, clinical oncology has greatly benefited from rapid progress in molecular testing, particularly next-generation sequencing (NGS). NGS provides higher analytical sensitivity and could simultaneously analyze numerous target genes and pathways in cancer [6]. Identification of driver mutations in tumors provides an opportunity to guide the selection of anticancer drugs and allows more precise targeted cancer therapy [7]. RNA sequencing quantifies the level of gene expression at the transcriptional level, and could detect sequence variants [8], and has been used as a new tool to prospectively test somatic mutations and germline variants. Integrating information obtained with DNA whole exome-sequencing (DNA–WES) and RNA sequencing could improve detection of cancer driver mutations [9].

Next-generation sequencing in combination with patient-derived xenograft (PDX) models (developed by LIDE Biotech) has been used to develop personalized treatment for melanoma [10]. PDXs allow preclinical evaluation of treatment effects [11], which, unlike cell line–derived tumor models, retain mutationally heterogeneous tumor cell populations similar to that in individual patients [12]. PDX models may also effectively mimic the treatment response of the parental tumor and can provide clues for selecting therapeutic target and regimen [13, 14]. Major obstacles in using conventional PDXs to develop personalized treatment plan for individual patients include low tumor engraftment rate and long period of time. PDX modeling is also expensive, technically cumbersome, and requires a large amount of tumor tissue [15]. Mini-PDX is a platform with reduced complexity and faster result turn-around. It is a promising tool where tumor cells from patients maintain tumorigenicity and are then inoculated into immunocompromised mice via a special capsule to establish tumor xenografts.

In the present study, we evaluated mini-PDX models combined with NGS (DNA–WES and RNA sequencing) analysis of metastatic tissue samples to guide personalized treatment in a patient with metastatic duodenal adenocarcinoma.

## Methods

### DNA–WES sequencing

DNA was extracted from fresh metastatic tumor tissues using a QIAamp DNeasy blood and tissue kit (Qiagen, Valencia, CA, USA). Raw WES reads were aligned to the reference genome (hg19) using the BWA-MEM aligner and further re-mapped for error correction and re-calibration using the Assembly Based ReAligner (ABRA) [16]. Polymerase chain reaction (PCR) duplications were removed by Picard software. The quality control metrics included coverage distribution, sequencing error and insert size estimation. Somatic mutations and genomic amplification/deletion were then detected from BAM files. Somatic single nucleotide variants (SNVs) were identified by MuTect (v1.17) with peripheral blood. Insertion-deletion polymorphisms (INDELs) were identified using PINDEL (V0.2.4). Additional filtering steps were used to reduce background noise. The functional impact of passed mutations was annotated by SnpEff3.0. The copy number variation (CNV) regions were identified by Control-FREEC (v9.4) [17] with the following parameters: window = 50,000 and step = 10,000. Gene fusions were detected through an in-house pipeline.

### RNA sequencing

Total RNA was isolated using the RNeasy^®^ Mini Kit (Qiagen), and the quality of RNA sequencing paired-end reads was assessed with FastQC. Reads were then mapped to the hg19 genome with the STAR RNA sequencing aligner [18]. Gene fusions at the transcript level were detected by STAR-fusion. Gene expression level was estimated with RSEM.

### Mini-PDX assay

**Fig. 1:**
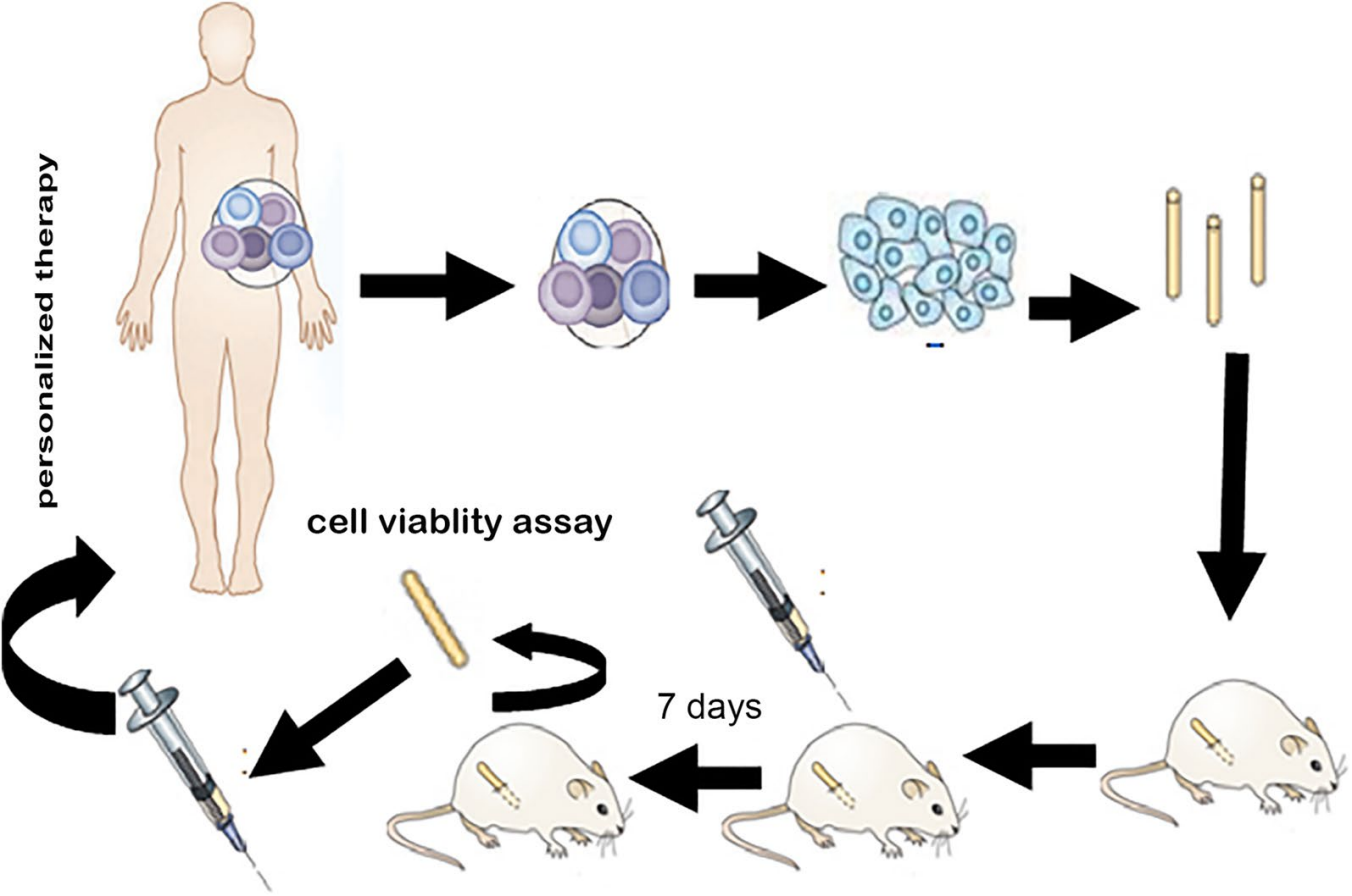
General schema of mini-PDX models

Mini-PDX assay was carried out using the OncoVee^®^ Mini PDX kit (LIDE Biotech Co., Ltd, Shanghai, China) (Fig. 1). Briefly, metastatic tumor samples were harvested, and then washed with Hanks’ balanced salt solution (HBSS) to remove non-tumor tissue and necrotic tumor tissue in a biosafety cabinet. The tumor samples were minced, followed by incubation with collagenase at 37 °C for 1–2 h. The cells were collected and blood cells and fibroblasts were then removed. The cell suspension was transferred to HBSS-washed capsules made of hollow fiber membrane with a pore size allowing passage of molecules less than 500 kDa. The fiber system delivered media to the cells in a manner similar to the delivery of blood through the capillary networks in vivo.

SCID mice (6–8 weeks of age) were housed and monitored in a SPF animal facility. For subcutaneous implantation, a small skin incision was made and the special capsule was inserted through the subcutaneous tissue. One day after tumor cell inoculation, tumor-bearing mice were randomized to receive vehicle, S-1 alone [10 mg/kg, orally administered (po), daily], oxaliplatin alone [5 mg/kg, intravenously (iv), every 4 days], irinotecan alone (50 mg/kg, iv, every 4 days), capecitabine alone (400 mg/kg, po, daily), nab-paclitaxel alone (20 mg/kg, iv, every 4 days), everolimus alone (10 mg/kg, po, daily), cisplatin (5 mg/kg, iv, every 4 days) in combination with S-1 (10 mg/kg, po, daily) or gemcitabine (60 mg/kg, iv, every 4 days). Seven days later, the capsules with tumor cells were removed. Cell viability was evaluated using a CTG assay.

### Statistical analysis

Statistical analyses were performed using SPSS version 16.0 (SPSS Inc., Chicago, IL, USA). One-way ANOVA followed by posthoc analysis was used for comparisons. *P* < 0.05 was considered statistically significant.

## Results

### Clinical history

**Fig. 2:**
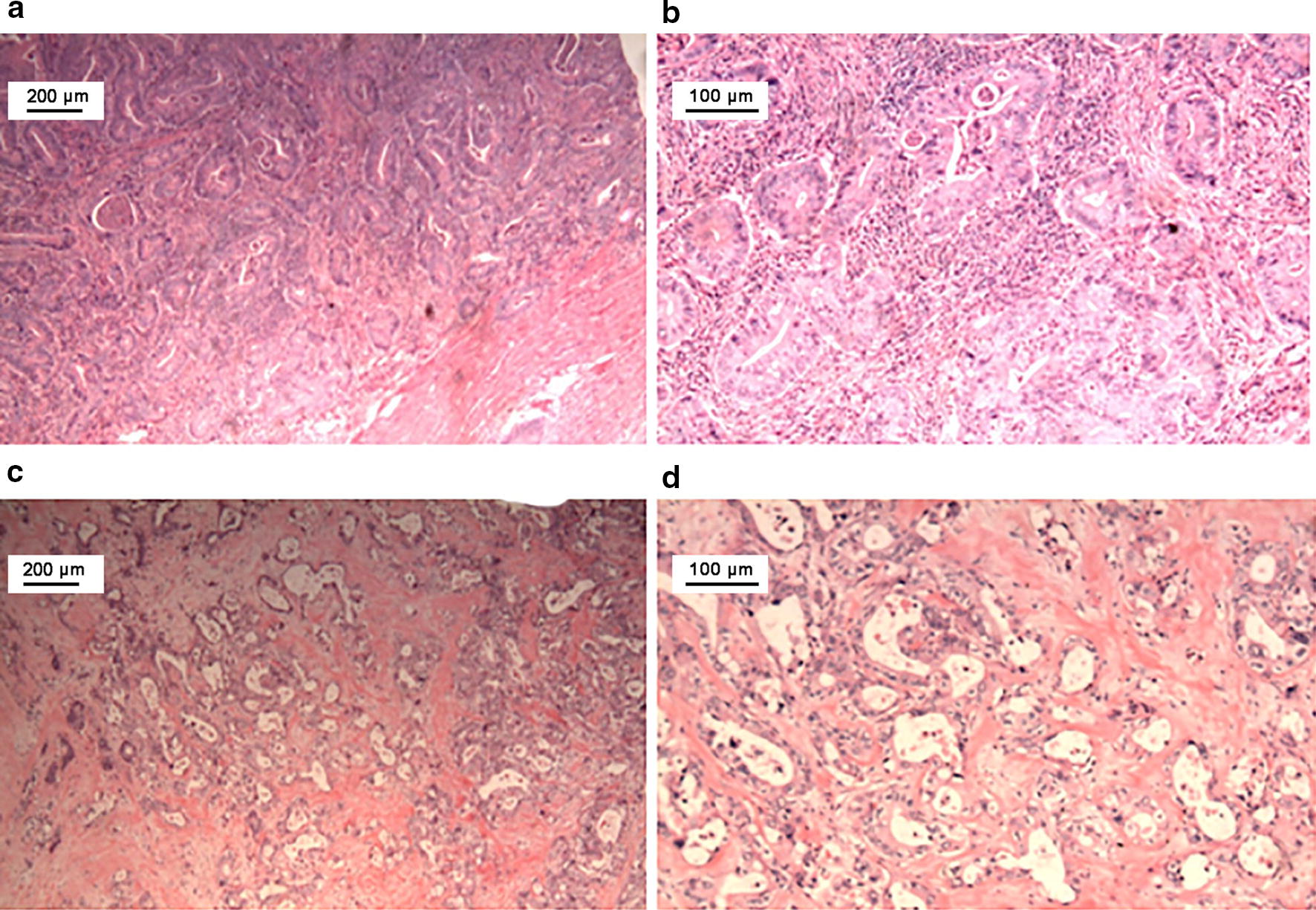
Histopathologic examination of the primary and metastatic duodenal adenocarcinoma by hematoxylin–eosin staining. **a**, **b** Microscopic examination reveals a moderately differentiated adenocarcinoma penetrating the visceral peritoneum and invading the pancreas. **c**, **d** Microscopic examination shows adenocarcinoma in the soft tissue of the superior mesenteric artery. **a**, **c**: ×40; **b**, **d**: ×100

**Fig. 3:**
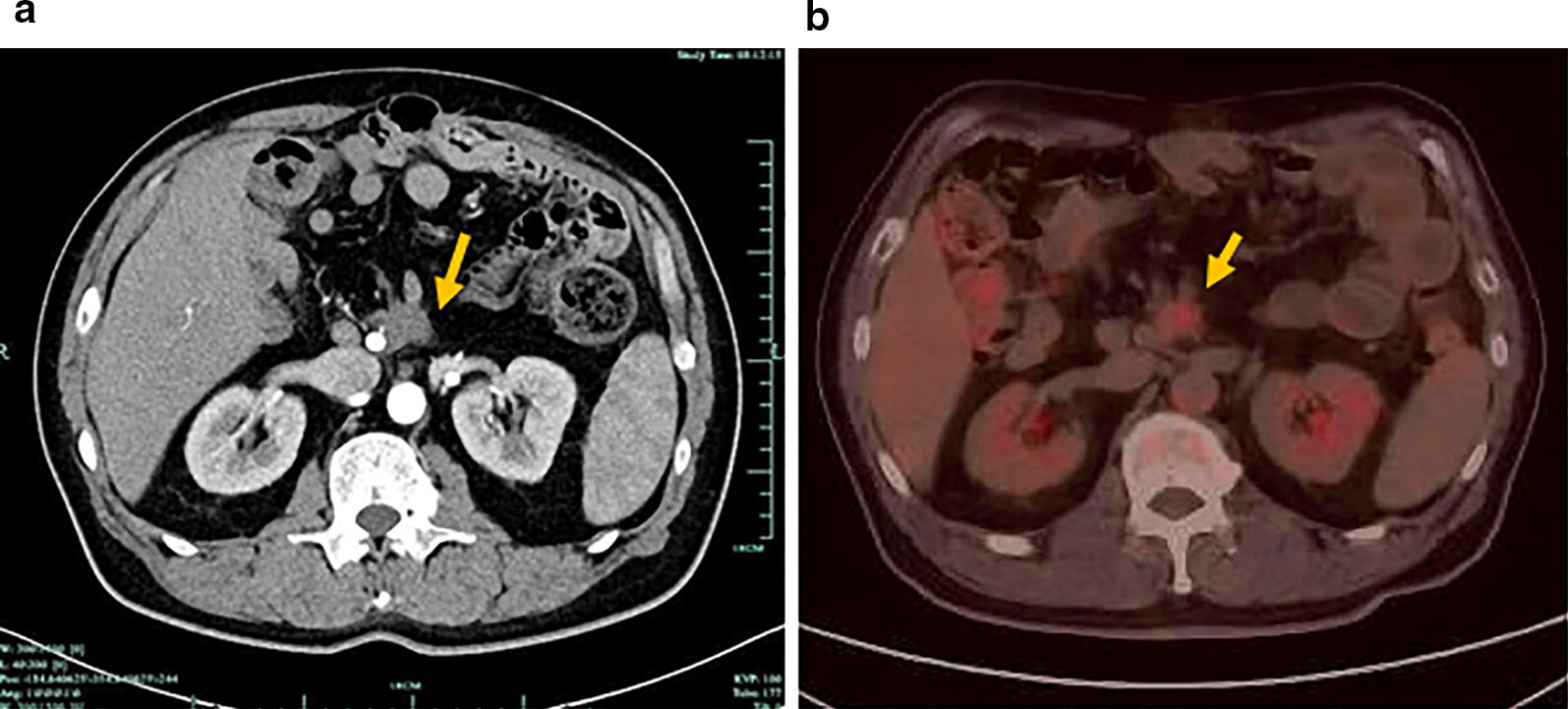
Computed tomography (CT) and PET/CT images of the metastatic lesion from a patient with duodenal adenocarcinoma. **a** CT scan shows the metastatic lesion (arrow) in the root of superior mesenteric artery and below transverse mesocolon. **b** PET/CT demonstrates malignant disease with FDG uptake (arrow) in the retroperitoneum

A 57-year-old man underwent a pancreaticoduodenectomy for duodenal adenocarcinoma in February 2014. The tumor penetrated the visceral peritoneum and invaded the pancreas with nodal involvement (pT4N1M0, stage IIIA, Fig. 2a). Starting from 1 month after the surgery, he received empirical treatment with 6 cycles of gemcitabine plus S-1 (GS) (gemcitabine 1000 mg/m^2^ on days 1 and 8, and oral S-1 60 mg twice daily on days 1–14, repeated every 3 weeks). A repeat CT scan in December 2016 revealed a lesion 2.2 cm in diameter in the root of the superior mesenteric artery and below the transverse mesocolon (Fig. 3a). PET/CT scan showed enhanced FDG uptake (Fig. 3b). The metastatic lesion was surgically removed and pathologically confirmed as metastatic duodenal adenocarcinoma in December 2016 (Fig. 2b).

**Fig. 4:**
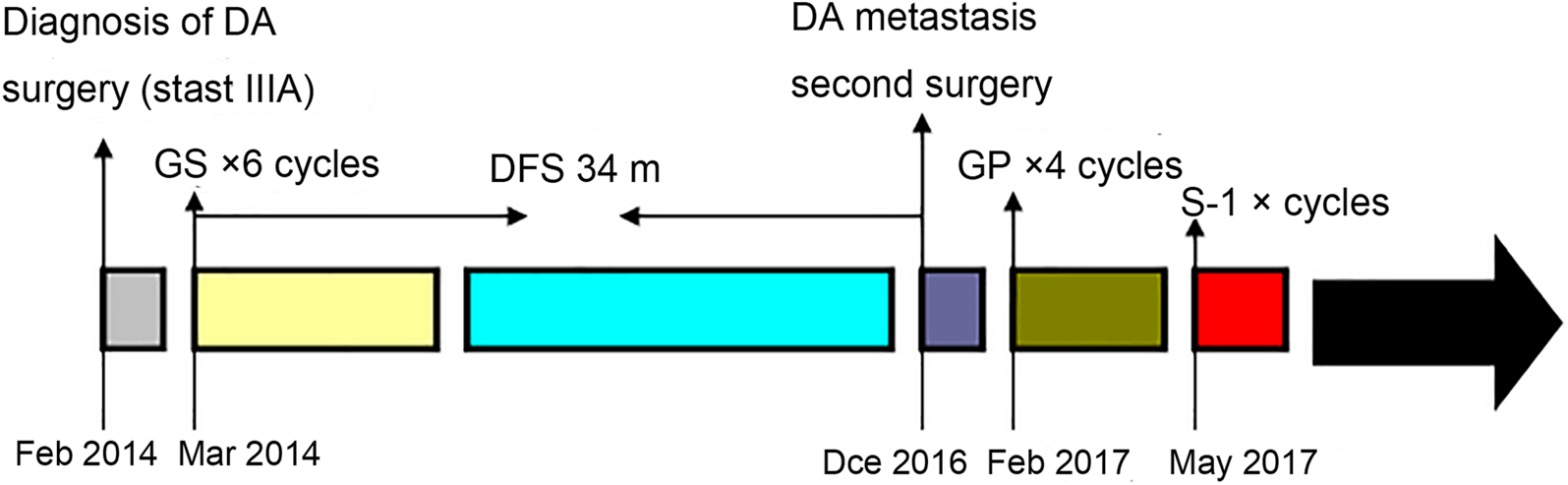
Flowchart summarizing treatments provided to the patient

Based on the results of the mini-PDX assessment, the patient was given a chemotherapy regimen that consisted of intravenous gemcitabine (1000 mg/m^2^) and cisplatin (25 mg/m^2^) on day 1 and 8, given every 3 weeks. The treatment was terminated after 4 cycles due to drug-related toxicities, including grade 3 nausea and fatigue. In May 2017, the patient was treated with 3 cycles of S-1 treatment (60 mg, po, twice daily for 14 consecutive days every 3 weeks). After the second surgery, the patient had completed adjuvant chemotherapy in February 2017. The last follow-up (9 months later) showed did not reveal tumor burden. The treatments and responses are summarized in Fig. 4.

### Mini-PDX assay

**Fig. 5:**
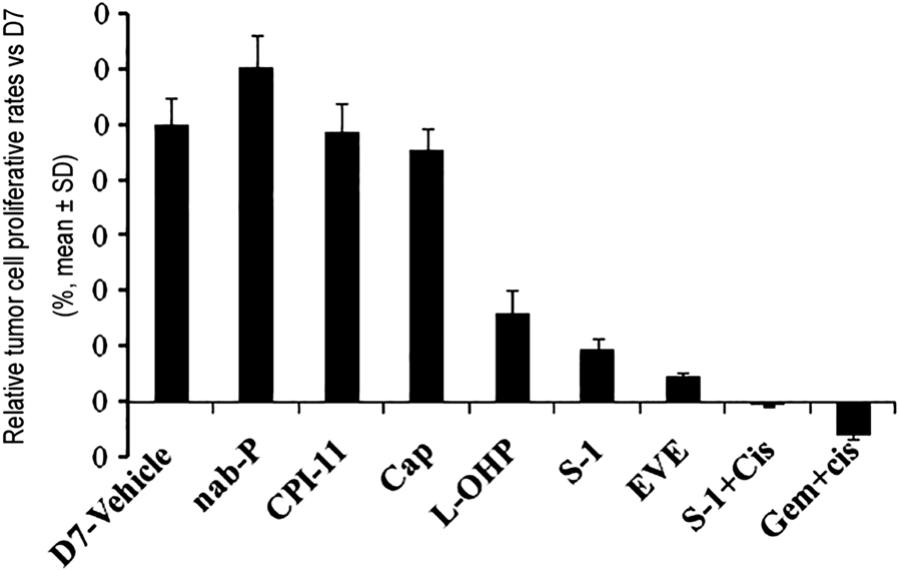
Mini PDX responses to chemotherapeutic and targeted regimens. Growth of mini-PDX from the patient in mice treated with vehicle, irinotecan, oxaliplatin, nab-paclitaxel, capecitabine, everolimus, S-1, gemcitabine plus cisplatin and S-1 plus cisplatin. One day after tumor cell inoculation, drug treatments commenced. Data show relative tumor cell proliferative rates versus the day 7 vehicle group

Mini-PDX assays showed that compared with the vehicle control on day 0, cell viability was decreased by 12.0% with S-1 plus cisplatin, and by 0.8% with gemcitabine plus cisplatin. The anti-proliferation rate with oxaliplatin, S-1, and everolimus was 67.6%, 80.3%, and 90.7%, respectively on day 7 (Fig. 5). Cell viability in the nab-paclitaxel, irinotecan and capecitabine group was not significantly different from the vehicle control (*P *> 0.05). No significant differences in body weight were observed among different treatment groups.

### DNA–WES and RNA sequencing analysis

DNA–WES and RNA sequencing were applied to a panel of 450 relevant cancer genes in metastatic duodenal adenocarcinoma tissues. Targeted gene sequencing was achieved in both tumor and normal samples with a mean coverage of 1728X and 431X, respectively. More than 85% of the reads were mapped to regions that the panel targeted or flanked in both tumor and normal samples. We identified the following somatic genetic changes in the metastatic tumor: *KRAS* mutation (A146T), *APC* (N1455Kfs*2), *TP53* (C229Yfs*10), *CXCR4* (V160I), *TERT* amplification, *MCL1* amplification and *RICTOR* amplification. Furthermore, we detected the following pharmacogenomic biomarkers: cisplatin (*GSTP1* rs1695 A/A), fluorouracil (*TPMT* rs1142345 A/A), and irinotecan (*UGT1A1* rs8175347 6/6; *SLCO1B1* rs2306283 G/A).

To confirm these mutations, we performed paired-end RNA sequencing of the metastatic sample to a depth of 52 million reads. Ninety-five percent of the reads (49 M) were aligned to the human genome (Additional file 1: Tables S1, S2). Of the aligned reads, 22.82% (12 M) were protein coding (Additional file 1: Figures S1, S2). Consistent with the results of WES, we identified the following somatic mutations in the transcriptomes of the metastatic tumor tissue: *KRAS* mutation (A146T), *TP53* (C229Yfs*10) and *RICTOR* amplification.

## Discussion

Currently, little data is available to guide rational choice of chemotherapeutic regimen for duodenal adenocarcinoma. The current practice at many centers is to treat duodenal adenocarcinoma patients with oxaliplatin-based chemotherapy similar to that for colorectal adenocarcinoma [2]. Gemcitabine-based adjuvant chemotherapy was offered to eligible patients with peri-ampullary duodenal adenocarcinoma in the ESPAC-3 trial [19]. In vivo models closely mimicking the biology of duodenal adenocarcinoma in patients are urgently needed to reliably determine optimal drug sensitivities of individual cancers for personalized therapy. PDX models of certain cancers closely mirror the drug response in patients compared with other models [20]. However, PDX models have several drawbacks that limit their clinical application. Generation of PDXs is only modestly successful and requires a large amount of tumor tissue which can usually only be obtained from surgically resected tumors. Furthermore, PDX models require 4–8 months to identify active therapies for a particular cancer patient. Elapse of time between tumor engraftment in mice and initiating treatment for patients is a limiting factor for real-time personalized medicine applications [12, 21]. To overcome these limitations, a new technology known as the mini-PDX model has emerged to guide selection of chemotherapeutic drugs. Mini-PDX models only require a small number of tumor cells and allow rapid analysis of drug sensitivity with a median latency of 7 days; thus, duodenal adenocarcinoma patients can receive personalized chemotherapy in a clinically relevant time frame. Previous studies have demonstrated a potentially strong agreement between mini-PDX- and PDXs-based prediction of drug sensitivity (overall response agreement: 89%) in a variety of solid tumors including lung cancer, pancreatic cancer, and gastric cancer. Importantly, the drug sensitivity pattern of mini-PDX can recapitulate responses in patients from which they were derived.

The median disease-free survival of duodenal adenocarcinoma patients with lymph node metastasis who underwent radical resection is 18–25 months [22, 23]. If recurrence develops, the survival time is only 5.4 months [22]. However, the disease-free survival of our patient who received GS as an adjuvant chemotherapeutic regimen was 34 months. Sensitivity of the cancer tissues to gemcitabine and S-1 in the mini-PDX experiments seems to be consistent with clinical response to the GS regimen in this patient. Following a second resection, the patient received gemcitabine and S-1-based chemotherapy according to the mini-PDX results. Therefore, mini-PDX is suitable for selecting optimal regimens for personalized therapy clinically. However, further studies with a large patient cohort are required.

DNA–WES and RNA sequencing identified patients with *KRAS* mutation and *RICTOR*-amplified tumors. *KRAS* mutation correlates significantly with late stage and poor tumor differentiation in duodenal adenocarcinoma and *KRAS*-mutated persons are more likely to experience distant relapse [24]. To date, there are no effective anti-Ras strategies. Activated KRAS induces a complex signaling network, including the PI3K/AKT/mTOR pathway. Due to difficulty in targeting KRAS directly, targeting downstream RAS effector signaling pathways are currently under clinical evaluation [25, 26]. RICTOR plays an important role in the PI3K/AKT/mTOR signaling pathway, which is one of the most commonly activated pathways in human cancer [27]. RICTOR is an essential component of the mTORC2 complex and upregulation of mTORC2 activity in turn regulates cell growth, metabolism, and survival [28]. It is also an important component of feedback loops and cross-talk in PI3K–AKT–mTOR signaling. *RICTOR* amplification is a rare, but recurrent somatic alteration in solid tumors [29]. Targeting mTOR to inhibit RICTOR may be a potential therapeutic strategy [30]. In the mini-PDX model, *RICTOR* amplified duodenal adenocarcinoma demonstrated sensitivity to mTORC1 inhibitor everolimus. However, inhibition of mTORC1 leads to an increase in AKT phosphorylation due to feedback loops that allow continued activity of mTORC2 [31]. The dual mTORC1/2 inhibitor, AZD2014, showed a more potent inhibitory effect on *RICTOR*-amplified tumors than everolimus [29].

Pro-survival protein myeloid cell leukemia 1 (*MCL1*) amplification was identified by DNA sequencing in this patient. MCL1, one of the anti-apoptotic BCL-2 family members, is overexpressed in many human cancers [32], and in several key oncogenic pathways to sustain cancer cell survival and resist cancer treatment. Repression of MCL1 exerts cytotoxic effects in tumor cells [33], indicating that MCL1 is a promising target for the treatment of a wide range of tumors. S63845, a small molecule that specifically binds with high affinity to the BH3-binding groove of MCL1, exerts anti-tumor activity in MCL1-dependent cancer cells in vitro and in vivo, while sparing normal tissues at efficacious doses, which represents a potential breakthrough in cancer therapy [34].

## Conclusions

Mini-PDX models in combination with DNA–WES/RNA analysis as an effective platform could be used to optimize the clinical management of metastatic duodenal adenocarcinoma.

## Additional file

**Additional file 1: Table S1.** Quality control metrics of targeted sequencing data. **Table S2.** Quality control metrics of RNA-seq data. **Figure S1.** Average coverage (Log10 transformed) of targeted genes with data deduplication. Coverage of mutant genes were shown in grey bar, error bars indicted standard deviation of sequencing depths for each gene. Coverage of all genes sequenced were shown in red line. **Figure S2.** Fraction of reads that mapped to coding, intergenic, ribosomal, intronic, UTR and unmapped reads for metastasis RNA-seq.
