# Supplementary material for: Personalized treatment based on mini patient-derived xenografts and WES/RNA sequencing in a patient with metastatic duodenal adenocarcinoma
Source: Cancer Commun (Lond). 2018 Aug 23;38:54. doi: 10.1186/s40880-018-0323-y (PMC6108145; doi:10.1186/s40880-018-0323-y)
Supplement: Supplementary file 1 — Additional file 1: Table S1. Quality control metrics of targeted sequencing data. Table S2. Quality control metrics of RNA-seq data. Figure S1. Average coverage (Log10 transformed) of targeted genes with data deduplication. Coverage of mutant genes were shown in grey bar, error bars indicted standard deviation of sequencing depths for each gene. Coverage of all genes sequenced were shown in red line. Figure S2. Fraction of reads that mapped to coding, intergenic, ribosomal, intronic, UTR and unmapped reads for metastasis RNA-seq. [file 40880_2018_323_MOESM1_ESM.docx]

Table S1 Quality control metrics of targeted sequencing data

| DNA-seq statistics | Metastatic tumor sample | Normal Blood sample |
| --- | --- | --- |
| Total paired reads | 131,511,308 | 23,179,706 |
| Aligned reads | 129,405,376 | 23,020,766 |
| Aligned reads(%) | 98.4% | 99.3% |
| Total bases | 9,966,484,989 | 1,757,394,414 |
| Aligned bases | 9,766,788,744 | 1,750,394,917 |
| Aligned bases(%) | 98.0% | 99.6% |
| Seq coverage^a^ | 4806 | 479 |
| deduped coverage | 1728 | 432 |
| On target(%) | 69.58 | 71.63 |
| On flanking(%) | 17.13 | 17.09 |
| Off target(%) | 13.29 | 11.28 |
| Seq error-rate | 0.0045 | 0.0039 |
| Insert size estimated | 224.49 | 223.87 |

^a^ sequence coverage before de-duplication.

Table S2 Quality control metrics of RNA-seq data

| RNA-seq statistics | Metastatic tumor sample |
| --- | --- |
| Total reads | 52,843,454 |
| Aligned reads | 49,785,763 |
| Aligned reads(%) | 94% |
| Total bases | 7,923,888,724 |
| Aligned bases | 7,375,518,110 |
| Aligned bases(%) | 93% |
| Ribosomal bases | 211,452,983 |
| Ribosomal bases(%) | 3% |
| Coding bases | 1,808,476,976 |
| Coding bases(%) | 25% |
| UTR bases | 1,737,703,765 |
| UTR bases(%) | 24% |
| Intronic bases | 494,191,043 |
| Intronic bases(%) | 7% |
| Intergenic bases | 3,125,511,437 |
| Intergenic bases(%) | 42% |

Figure S1. Average coverage (Log10 transformed) of targeted genes with data deduplication. Coverage of mutant genes were shown in grey bar, error bars indicted standard deviation of sequencing depths for each gene. Coverage of all genes sequenced were shown in red line.


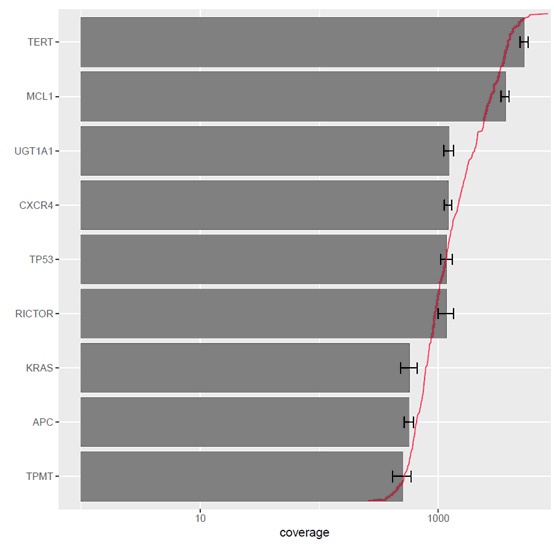


Figure S2. Fraction of reads that mapped to coding, intergenic, ribosomal, intronic, UTR and unmapped reads for metastasis RNA-seq.
